# Protein biomarkers of inflammation in women with SLE and their newborn children

**DOI:** 10.1136/lupus-2026-002001

**Published:** 2026-09-29

**Authors:** Christina Gerstner, Emma Kroon, Tilen Tršelič, Gudny Ella Thorlacius, Claudia Arasa, Sophia Björkander, Rui Da Silva Rodrigues, Eva Sverremark-Ekström, Marie Wahren-Herlenius, Malin Hedlund

**Affiliations:** 1 Department of Medicine Solna, Division of Rheumathology, Karolinska Institutet, Stockholm, Sweden; 2 Center for Molecular Medicine, Karolinska University Hospital, Stockholm, Sweden; 3 Department of Molecular Biosciences, The Wenner-Gren Institute, Stockholm University, Stockholm, Sweden; 4 Department of Clinical Science and Education, Karolinska Institutet, Stockholm, Sweden; 5 Department of Clinical Immunology and Transfusion Medicine, Karolinska University Hospital, Stockholm, Sweden; 6 Department of Clinical Science, Broegelmann Research Laboratory, University of Bergen, Bergen, Norway; 7 Department of Women's and Children’s Health, Karolinska Institute, Stockholm, Sweden

**Keywords:** Autoantibodies, Autoimmune Diseases, Inflammation, Lupus Erythematosus, Systemic, Interferon Type I

## Abstract

**Objectives:**

In utero exposure to maternal anti-Ro/Sjögren’s syndrome antigen A (SSA) and anti-La/Sjögren’s syndrome antigen B (SSB) autoantibodies activates the fetal type I interferon (IFN) system. Women with SLE often carry a broader range of autoantibodies beyond anti-Ro/SSA and anti-La/SSB, like for example, anti-double stranded DNA (anti-dsDNA), anti-centromere and anti-nucleosome antibodies. We aimed to assess IFN activation and the expression of other immune-related proteins in pregnant women with SLE—with or without anti-SSA/SSB or anti-dsDNA autoantibodies—and in their newborns. We also aimed to investigate the impact of maternal immunomodulatory treatment on these responses.

**Methods:**

Serum samples from 22 women with SLE and 22 healthy pregnant women, along with their newborns, were analysed using proximity extension assay technology to quantify 92 immune-related proteins.

**Findings:**

Women with SLE had differential expression of 39 out of 92 proteins compared with healthy controls. Several proinflammatory cytokines and proteins involved in SLE pathogenesis were significantly upregulated in both women and their newborns. A couple of IFN-regulated proteins were significantly increased in anti-SSA/SSB-positive and anti-dsDNA-positive mother-child pairs, respectively. Still, IFN scores were generally elevated in pregnant women with SLE regardless of their autoantibody status. However, neonatal IFN scores did not significantly differ based on maternal autoantibody status or treatment.

**Conclusion:**

Women with SLE and their newborn children had increased expression of a range of proinflammatory biomarkers irrespective of their anti-SSA/SSB or anti-dsDNA autoantibody status. Irrespective of the increased expression of several IFN-regulated proteins in anti-SSA/SSB-positive and anti-dsDNA-positive mother-child pairs, the presence of these autoantibodies alone did not significantly influence the level of IFN activation in neither the mothers nor the newborns exposed to these autoantibodies in utero.

**WHAT IS ALREADY KNOWN ON THIS TOPIC**

- Maternal anti-Ro/Sjögren’s syndrome antigen A (SSA) and anti-La/Sjögren’s syndrome antigen B (SSB) autoantibodies can activate the neonatal type I interferon (IFN) system, and their transplacental transfer may result in congenital heart block.
- SLE is associated with a broad spectrum of autoantibodies that may influence IFN activation and the expression of other proinflammatory proteins.

**WHAT THIS STUDY ADDS**

- This study demonstrates increased expression of multiple proinflammatory proteins—primarily cytokines and chemokines—in pregnant women with SLE and their newborns compared with healthy controls.
- IFN scores are elevated in pregnant women with SLE regardless of anti-SSA/SSB or anti-double stranded DNA (anti-dsDNA) autoantibody status, respectively, compared with pregnant healthy controls.

**HOW THIS STUDY MIGHT AFFECT RESEARCH, PRACTICE OR POLICY**

- The findings in this study highlight the broad immune activation in SLE pregnancies beyond traditional autoantibody responses.
- Further studies are needed to determine the clinical relevance of these immune differences and their potential implications for clinical practice.
- Future studies should also explore whether maternal immunomodulatory treatment is associated with differences in neonatal immune profiles and whether these findings have any relevance for pregnancy outcomes.

## Introduction

 SLE is a complex, chronic autoimmune disease involving multiple organ systems.^1^ It typically affects women of childbearing age and arises from an interplay of genetic and environmental factors.^2^ A hallmark of systemic autoimmune diseases, like SLE and Sjögren’s disease (SjD), is the presence of circulating autoantibodies targeting nuclear and intracellular antigens, including double stranded DNA (dsDNA), Ro/Sjögren’s syndrome antigen A (SSA) and La/Sjögren’s syndrome antigen B (SSB).^3 4^ All of the autoantibodies, especially when present in immune complexes with their cognate antigens, are viewed as essential mediators of SLE pathology and have been associated with activation of the type I interferon (IFN)-system.^5^ The upregulation of IFN-stimulated genes, commonly referred to as the ‘IFN signature’, can be quantified by an IFN score.^6 7^

Patients with SLE are at an increased risk for pregnancy-related complications, such as pre-eclampsia, preterm delivery, fetal growth restrictions or even fetal loss.^8^ Neonatal lupus erythematosus with a congenital heart block (CHB) occurs in 2–3% of pregnancies in women with anti-Ro/SSA and/or La/SSB antibodies and is closely linked to the transfer of these autoantibodies across the placenta to the fetus.^9–11^ Many of the mothers of affected infants are diagnosed with rheumatic diseases, most commonly SjD or SLE, while some have mild symptoms not fulfilling the diagnosis criteria or are even asymptomatic.^12 13^

Experimental studies have shown that in vitro exposure of cord blood immune cells from healthy pregnancies to sera containing anti-Ro/SSA and anti-La/SSB antibodies induces expression of IFN-regulated genes.^14^ In utero, fetuses exposed to these autoantibodies express IFNs and display activation of the type I IFN system, and their IFN scores correlate with those of their mothers.^14^ Notably, maternal immunomodulatory treatment has been reported to reduce neonatal IFN activation, sometimes to a greater extent than in the mother.^14^ However, the role of other immune-related proteins and their maternal-neonatal correlation remain less explored.

Women with SLE often carry additional autoantibodies beyond the ones against dsDNA, Ro/SSA and La/SSB, some of which may contribute to IFN activation. In the present study, we aimed to investigate the degree of IFN activation and levels of other immune-related proteins in pregnant women with SLE—subgrouped according to the presence of anti-Ro/SSA and/or anti-La/SSB as well as anti-dsDNA autoantibodies—and in their newborns. We also sought to investigate how maternal immunomodulatory treatment influences the presence of these proteins and the IFN activation in both mother and child.

## Materials and methods

### Patients and healthy controls

Serum samples from pregnant women and cord blood from their newborns were collected at delivery. Blood samples were processed immediately after childbirth, cryopreserved and stored at −80 °C until analysis. All 22 women with SLE and 22 healthy controls and their newborns were included at the Karolinska University Hospital, Stockholm, Sweden, between the years 2004 and 2014. Healthy control women were clinically healthy, with no history of autoimmune disease and not taking any medication. The age of the women at delivery was collected; no further clinical information of the healthy pregnancies was obtained. Women with SLE fulfilled the revised 1982 American College of Rheumatology criteria. Demographic and clinical information on the mothers with SLE diagnosis are summarised in tables 1 and 2. All newborns were born without any malformations and no cases of CHB were reported. Looking at other adverse pregnancy outcomes, like preterm birth and being small-for-gestational-age, two infants, both born to women positive for anti-Ro/La autoantibodies, were born at <37 weeks of gestational age (week 36+0 and 36+6, respectively). One infant born to a woman positive for anti-Ro/La autoantibodies was small-for-gestational-age (−2.15 SD).^15^ Data on gestational age, birth mode, sex, weight, height and head circumference were obtained for some of the neonates born to women with SLE (online supplemental table 1a,b). The study was approved by the Local Ethics Committee Stockholm, and the mothers gave written informed consent (Dnr: 03–417; 2006–1706-32).

**Table 1:** Demographic and clinical characteristics of included mothers grouped according to anti-SSA/SSB autoantibody status

	Mothers		
	Rheumatic disease		Healthy controls
	Anti-SSA/SSB+, n=11	Anti-SSA/SSB−, n=11	n=22
Age at delivery (years, mean±SD)	31.5±3.50	33.2±4.83	33.0±5.61
Rheumatic diagnosis			
SLE	10	10	0
SLE+SjD	1	0	0
SLE+APS	0	1	0
Medication at sampling (%)			n.a.
Plaquenil	5 (45.5)	3 (27.3)	
Prednisolone	6 (54.5)	5 (45.5)	
Azathioprine	0	2 (18.2)	
No medication	3 (27.3)	5 (45.5)	
Autoantibodies: Ro52/Ro60/La (%)		n.a.	n.a.
+/+/+	7		
+/+/−	1		
−/+/+	1		
−/+/−	2		
Other autoantibodies (%)			n.a.
β−2-glycoprotein	3 (27.3)	3 (27.3)	
Cardiolipin	0	3 (27.3)	
Centromere	1 (9.09)	1 (9.09)	
dsDNA	4 (36.4)	4 (36.4)	
Nucleosome	1 (9.09)	3 (27.3)	
Ribosomal P	1 (9.09)	0	
Ribonucleoprotein particle (RNP)	2 (18.2)	0	
Smith (Sm)	1 (9.09)	2 (18.2)	
Sm/RNP	1 (9.09)	1 (9.09)	

APS, antiphospholipid syndrome; SjD, Sjögren’s disease.

**Table 2:** Demographic and clinical characteristics of included mothers grouped according to anti-dsDNA autoantibody status

	Mothers	
	Rheumatic disease	
	Anti-dsDNA+, n=8	Anti-dsDNA−, n=14
Age at delivery (years, mean±SD)	33.3±3.24	31.8±4.71
Rheumatic diagnosis		
SLE	7	13
SLE+SjD	1	0
SLE+APS	0	1
Medication at sampling (%)		
Plaquenil	1 (12.5)	2 (14.3)
Prednisolone	4 (50.0)	2 (14.3)
Azathioprine	1 (12.5)	1 (7.14)
No medication	2 (25.0)	6 (42.9)
Other autoantibodies (%)		
anti-Ro52/Ro60/La	4 (50.0)	7 (50.0)
β−2-glycoprotein	2 (25.0)	1 (7.14)
Cardiolipin	2 (25.0)	1 (7.14)
Centromere	1 (12.5)	1 (7.14)
Nucleosome	2 (25.0)	2 (14.3)
Ribosomal P	1 (12.5)	0
RNP	0	2 (14.3)
Sm	1 (12.5)	2 (14.3)
Sm/RNP	1 (12.5)	1 (7.14)

APS, antiphospholipid syndrome; SjD, Sjögren’s disease.

### Protein biomarker analysis

Serum samples were analysed using the Olink Target 96 Immuno-Oncology Panel (Olink Biosciences, Sweden). The technology is based on proximity extension assays that in this case enable the simultaneous detection of 92 proteins associated with immuno-oncology (online supplemental table 2). Normalised protein expression values were provided for each protein.

### Bioinformatic and statistical analysis

For the Olink data, differentially expressed proteins were identified using base R by applying two-sided unpaired t-tests between groups. Proteins with a Log2 fold change (FC) >0.5 and p value <0.05 were considered enriched and those with a Log2 FC <−0.5 and p value <0.05 were considered depleted in volcano plots. For heat maps, a p value threshold of 0.05 was used and hierarchical clustering was performed using Euclidean distance on z-scored data. The R package ggplot2 was used to visualise heat maps and volcano plots. Correlation analysis of protein expression was performed by calculating Spearman’s rank correlation coefficient.

Data not analysed by R as described above were analysed with GraphPad Prism, V.10.2.3, software (Dotmatics, Boston, Massachusetts, USA). Comparison between two groups was made using Mann-Whitney U-test. Multiple comparison was performed using Kruskal-Wallis test followed by Dunn’s test. P values <0.05 were considered significant. Information on which statistical method was applied is stated in the respective figure legends.

### IFN score calculations

IFN scores were calculated as Z-scores as previously described using the expression of six type I IFN-induced proteins: CXCL10, CXCL11, tumour necrosis factor-related apoptosis-inducing ligand (TRAIL), lysosome-associated membrane glycoprotein 3 (LAMP3), programmed cell death ligand 1 (PD-L1) and PD-L2.^14 16–21^ Briefly, data from the healthy control group (mean and SD for each of the six proteins) were used to standardise expression levels of each protein for each subject. IFN scores for each mother and newborn were then calculated by summarising the standardised expression levels of all six proteins. The mean IFN score in SLE mothers was 5.17, with a range from −2.61 to 15.2 and −3.64×10^–7^, with a range from −6.05 to 13.3 for healthy controls. In newborns, the IFN score was 2.15, with a range from −5.01 to 15.4 for the ones born to women with SLE and −1.82×10^–7^, with a range from −8.24 to 6.58 for neonates born to healthy mothers.

### Patient and public involvement

Patients or the public were not involved in the design, conduct, reporting or dissemination plans of our research.

## Results

### Protein biomarkers in women and their newborns

High-multiplex immunoassay profiling was performed on sera collected at delivery from 22 women with SLE and 22 healthy controls, along with cord blood samples from their 44 newborns. Protein levels were measured using the Olink Immuno-Oncology panel, covering 92 immune-related proteins. Principal component analysis (PCA) of the protein expression data revealed that most variation stemmed from the sample origin, with maternal samples clustering separately from neonatal samples (figure 1A). However, women with SLE were distinctly separated from healthy controls in the second dimension (principal component 2, PC2), a difference less evident in neonates.

**Figure 1:**
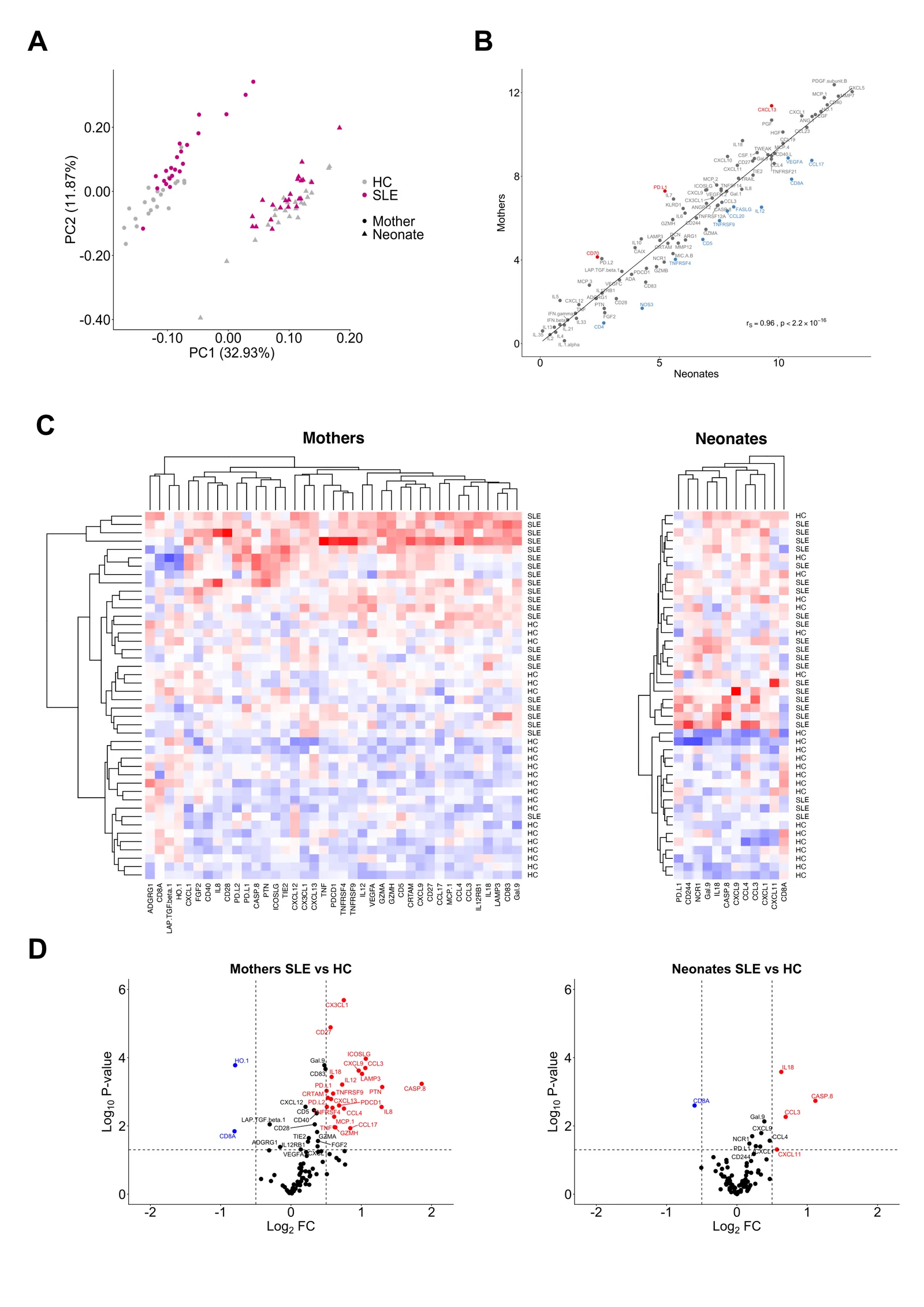
Distribution of protein biomarkers in mothers and their newborns. (**A**) Principal component analysis (PCA) score plot of maternal and neonatal samples from women with SLE (pink) and HC (grey), projected onto the first two principal components (PC1 and PC2). Circles represent maternal samples; triangles represent neonatal samples. (**B**) Scatter plot showing the correlation of average protein expression levels between mothers and their neonates (Spearman’s rank correlation coefficient r_S_=0.96, p<2.2×10⁻¹⁶). Proteins with Log2 FC>0.5 (maternal enrichment) are highlighted in red, and those with Log2 FC<−0.5 (neonatal enrichment) are shown in blue. (**C**) Heatmaps displaying all proteins significantly differentially expressed (p<0.05) between SLE and healthy mothers (left) and between their respective neonates (right). Each column corresponds to a protein, each row to an individual sample. Hierarchical clustering was performed using Euclidean distance on z-scored data. (**D**) Volcano plot of the differentially expressed proteins in SLE mothers versus healthy mothers (upper graph) and neonates born to SLE mothers versus neonates born to healthy mothers (lower panel). Significantly elevated proteins (Log2 FC<0.5) are coloured in red, significantly decreased proteins (Log2 FC<−0.5) are depicted in blue. FC, fold change; HC, healthy control.

Across the 92 proteins, we observed a strong and highly significant positive correlation between mean protein expression levels in mothers and their newborns (figure 1B, online supplemental table 3a). Several proteins were enriched in mothers compared with neonates, including CD70, PD-L1 and CXCL13 (Log2 FC >0.5). In contrast, proteins such as CD4, CD5, CD8A, nitric oxide synthase 3 (NOS3), TNF Receptor Superfamily Member 4 (TNFRSF4, OX40), TNF Receptor Superfamily Member 9 (TNFRSF9, 4–1BB), Fas ligand, vascular endothelial growth factor A (VEGF-A), CCL17, CCL20 and interleukin-12 (IL-12) were more abundant in neonates (Log2 FC <−0.5).

The PCA separation between SLE and control mothers was largely explained by the differential expression of 39 proteins (figure 1C, left heatmap). Hierarchical clustering based on these proteins grouped the SLE patients together, with most showing elevated expression levels compared with healthy controls. In neonates, 12 proteins were differentially expressed between those born to SLE mothers and those born to healthy controls (figure 1C, right heatmap). However, unsupervised clustering of these did not result in clear separation of the two groups.

### Protein biomarkers enriched in women with SLE and their newborns

Among the 39 proteins differentially expressed between SLE and healthy mothers, 23 were enriched at a Log2 FC >0.5, and two were downregulated at Log2 FC <−0.5 in the SLE group (figure 1D, left panel, online supplemental table 3b). Upregulated proteins included cytokines such as CX3CL1, CXCL9, CCL2 (monocyte chemoattractant protein 1, MCP-1), IL-12, IL-18 and tumour necrosis factor (TNF), as well as immune regulators like caspase-8 (CASP.8), inducible T-cell costimulator (ICOS) ligand, PD-1 (PDCD1), LAMP3, OX40 and 4–1BB. Downregulated proteins included CD8A and heme oxygenase 1 (HO-1).

In neonates, four of the 12 differentially expressed proteins—IL-18, CASP.8, CCL3 and CXCL11—were elevated at Log2 FC >0.5 in those born to SLE mothers, while CD8A was the only protein decreased at Log2 FC <−0.5 (figure 1D, right panel, online supplemental table 3b). Notably, four proteins—IL-18, CCL3, CASP.8 and CD8A—showed consistent differential expression in both SLE mothers and their newborns. As expected, levels of these proteins were significantly correlated between mother-child pairs, regardless of maternal SLE diagnosis or autoantibody status (online supplemental figure 1A, data not shown). Interestingly, however, CASP.8 was the only protein found at statistically lower levels in neonates born to treated women compared with the ones born to untreated women. Looking in detail at the different treatment groups revealed that neonates born to women treated with hydroxychloroquine had the lowest CASP.8 levels (online supplemental figure 1B). These findings should, however, be interpreted with caution, as first the different treatment subgroups get rather small and second information on maternal disease activity was not available and may have contributed to the observed differences.

Comparison between anti-SSA/SSB-positive and anti-SSA/SSB-negative SLE mothers revealed differences in the expression of two proteins: arginine-1 found to be decreased and CCL19 to be increased in SSA/SSB-positive women (figure 2A, left panel). In neonates exposed versus unexposed to anti-SSA/SSB antibodies, we detected four proteins, namely arginine-1, CASP.8, adenosine deaminase and LIGHT/TNF superfamily member 14, to be decreased and one protein, CXCL10, to be increased in anti-SSA/SSB-exposed neonates (figure 2A, right panel).

**Figure 2:**
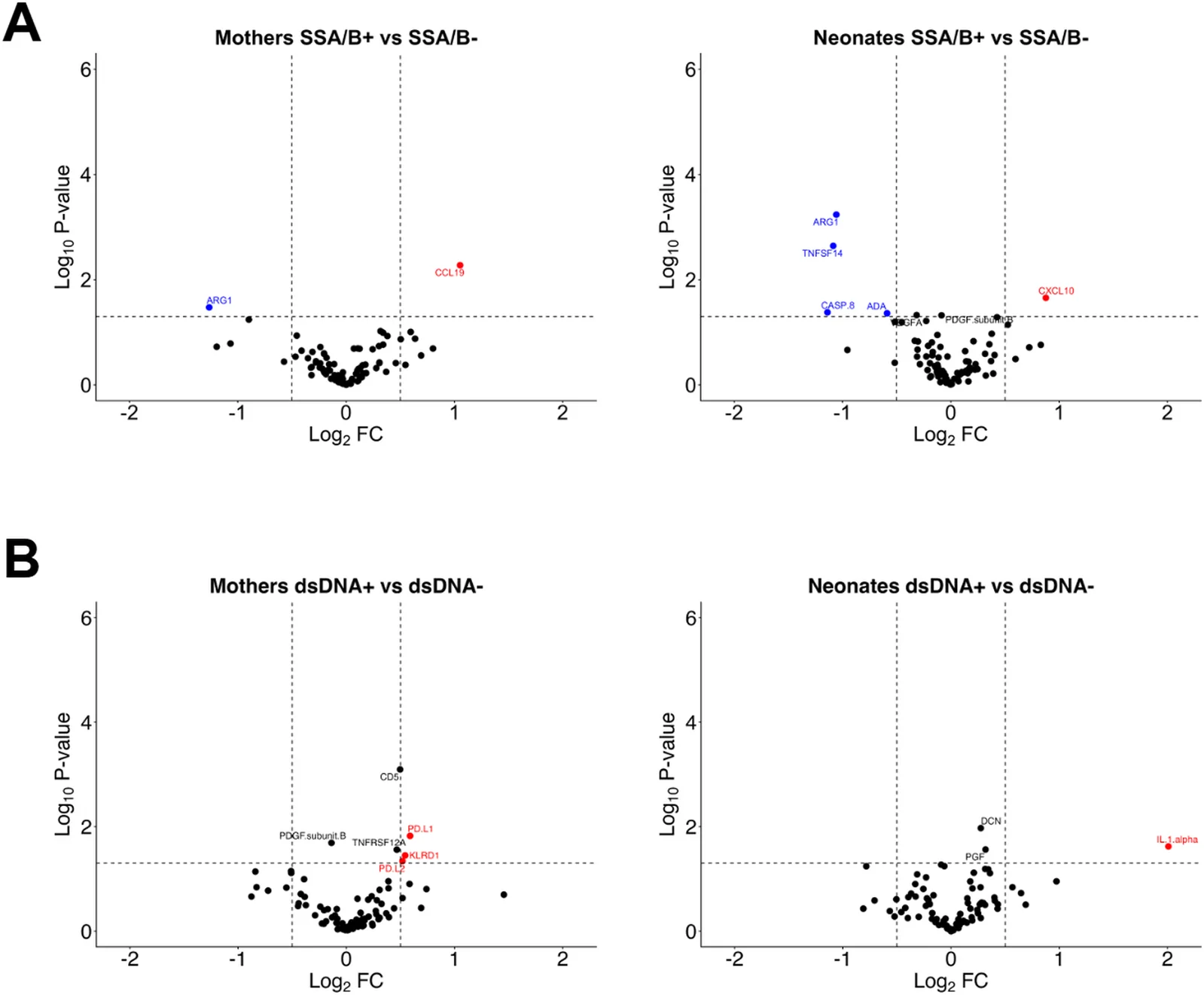
Differential protein expression by anti-SSA/SSB and anti-dsDNA autoantibody status. Volcano plots of protein levels in SLE mothers positive versus negative for **(A**) anti-SSA/SSB autoantibodies and **(B**) anti-dsDNA autoantibodies and their corresponding neonates. Proteins significantly upregulated (Log2 FC <0.5) in SSA/SSB-positive or dsDNA-positive individuals, respectively, are shown in red; downregulated proteins (Log2 FC <−0.5) are shown in blue. FC, fold change.

Comparing anti-dsDNA-positive and anti-dsDNA-negative SLE mothers on the other hand revealed differences in the expression of three proteins: PD-L1, PD-L2 and KLRD1 which all were increased in women with anti-dsDNA-antibodies (figure 2B, left panel). In neonates born to women with anti-dsDNA-antibodies IL-1α was the only protein found to be upregulated when compared with neonates born to women without anti-dsDNA antibodies (figure 2B, right panel).

### IFN score stratified by autoantibody profile

To understand how the expression of IFN-regulated genes related to the presence of autoantibodies, we calculated IFN scores based on the expression data of six type I IFN-induced proteins: CXCL10, CXCL11, TRAIL, LAMP3, PD-L1 and PD-L2. SLE mothers, regardless of anti-SSA/SSB and anti-dsDNA antibody status, respectively, exhibited significantly higher IFN scores than healthy controls (figure 3A,B and online supplemental figure 2A). Among neonates, IFN scores did not differ either between those born to SSA/SSB-positive versus SSA/SSB-negative mothers and dsDNA-positive versus dsDNA-negative mothers, respectively, nor between these and neonates born to healthy women (figure 3A,B and online supplemental figure 2A). Nevertheless, some anti-SSA/SSB autoantibody-exposed neonates showed modestly elevated IFN scores compared with controls.

**Figure 3:**
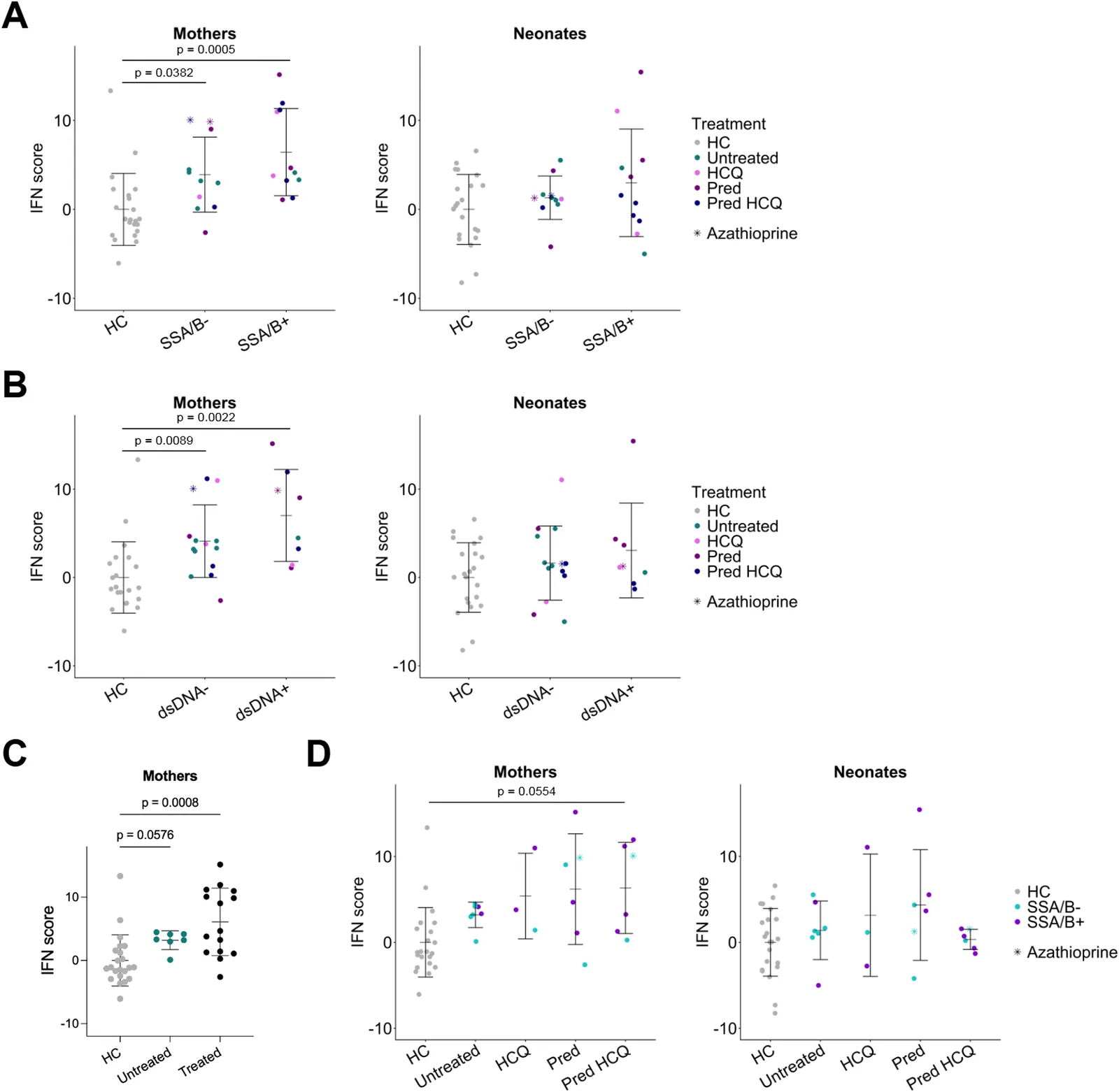
Type I IFN activation scores in relation to autoantibody status and treatment. IFN scores, derived from protein levels of CXCL10, CXCL11, TRAIL, LAMP3, PD-L1 and PD-L2, are plotted for mothers (left), divided by diagnosis into HC, anti-SSA/SSB-negative and anti-SSA/SSB-positive groups (A) or anti-dsDNA-negative and anti-dsDNA-positive groups (B), respectively, and their neonates (right), colour-coded for different treatment: grey=healthy controls; light blue=untreated; pink=HCQ; violet=Pred; dark blue=Pred + HCQ. (C) IFN scores plotted for mothers, grouped by treatment: HC (grey), untreated (green) and treated (black). (D) IFN scores plotted for mothers (left), grouped by treatment: healthy controls, untreated, HCQ, prednisolone (Pred) or Pred+HCQ and their neonates, grouped by maternal treatment as described above. Colour-coding reflects maternal diagnosis according to anti-SSA/SSB autoantibody status: grey=healthy control; blue=SSA/SSB-negative SLE; violet=SSA/SSB-positive SLE. Samples from the two mothers receiving azathioprine in combination with Pred or Pred+HCQ are marked with an asterisk (*). Statistical analysis was performed using Kruskal-Wallis test for overall comparison, followed by Dunn’s test to compare the different groups. P values <0.05 were considered significant and are stated in the plots. HC, healthy control; HCQ, hydroxychloroquine; IFN, interferon.

When correlating maternal and neonatal IFN scores, a trend towards significance (p*=*0.053) was seen for SLE mothers and their newborns with a Spearman’s rank coefficient of 0.42 (online supplemental figure 2B). A strong positive correlation (r_S_=0.65, p=0.015) was exclusively observed in anti-dsDNA-negative mother-child pairs, but not in healthy control pairs or any of the other autoantibody-dependent subgrouped mother-child pairs (online supplemental figure 2C,D).

### IFN score and immunomodulatory treatment

We next examined the effect of maternal immunomodulatory therapy on IFN scores. Women with SLE receiving treatment had significantly higher IFN scores than healthy controls (figure 3C,D). This was, however, not mirrored in the infants born to these women (online supplemental figure 2E). The group of SLE mothers receiving prednisolone in combination with hydroxychloroquine showed elevated IFN scores, although not significant, compared with healthy controls (figure 3D). Again, for the infants, despite increased IFN scores in some of those born to mothers treated with hydroxychloroquine or prednisolone, respectively, there was no difference between any of the groups (figure 3D). This might also explain why no correlation was seen between maternal and neonatal IFN scores in any of the treatment subgroups (online supplemental figure 2F). Given that data on maternal disease activity were not available, the observed differences should be interpreted with caution, as disease activity might influence both IFN scores and the need for immunomodulatory treatment, particularly corticosteroids.

## Discussion

In this study, we analysed immune-related protein profiles in pregnant women with SLE and healthy controls, along with their respective newborns. Several proteins were found to be differentially expressed between SLE and healthy mothers, as well as between their neonates. Notably, we found increased expression of several IFN-regulated proteins in anti-SSA/SSB-positive SLE mothers and their neonates as well as in anti-dsDNA-positive SLE mothers. Still, when assessing IFN pathway activation, we found elevated IFN scores in SLE mothers irrespective of the presence of anti-Ro/SSA and anti-La/SSB, as well as anti-dsDNA autoantibodies. Interestingly however, a positive correlation between maternal and neonatal IFN scores was only observed in dsDNA-negative mother-infant pairs.

Despite distinct clustering of maternal and neonatal samples in PCA, there was a strong overall correlation in the expression levels of most proteins between mother-newborn pairs. Some proteins, including CD70, PD-L1 and CXCL13, were enriched in maternal samples, while others such as CD4, CD5, CD8, NOS3 and VEGF-A were more abundant in neonates. The elevated expression of CD4, CD5 and CD8 in cord blood is consistent with previous findings showing increased T and B cell frequencies in neonates compared with adults.^22 23^ CD5^+^ B cells are particularly prevalent in cord blood and gradually decrease during childhood.^24 25^ Similarly, NOS3 and VEGF-A, both key regulators of placental and fetal vascular development,^26 27^ were enriched in neonates, in line with their known physiological roles.^28^

Among the 39 proteins upregulated in mothers with SLE, many were pro-inflammatory cytokines and chemokines (eg, MCP-1/CCL2, CCL3, IL-8, IL-12, IL-18, TNF), several of which are associated with IL-10 signalling. Although IL-10 itself was not elevated, its downstream effects have been shown to influence the pathogenesis of SLE by driving extrafollicular autoantibody production via a specialised subset of extrafollicular B helper T cells^29^ and an increased activity of the IL-10 pathway has been documented in murine models of SLE as well as in SLE patients.^30 31^ Increased expression of beta-chemokines (eg, CCL2, CCL3, CCL4), CXCL13, IL-12 and IL-18 in SLE mothers supports previous findings linking these molecules to disease activity and complications such as lupus nephritis and cutaneous lupus.^32–35^ The role as biomarkers for SLE and even as targets for new specific therapeutics has long been proposed for some cytokines.^36–38^ Another secreted protein that we found to be increased in women with SLE is pleiotrophin (PTN), a heparin-binding growth-associated molecule. Interestingly, PTN has been shown at significantly elevated levels in the plasma of patients suffering from rheumatoid arthritis, SjD and SLE with its expression being linked to high levels of IL-17 and the presence of anti-Ro/SSA antibodies.^39^

Other upregulated proteins in SLE mothers, such as granzyme H (GZMH), LAMP3 and CASP.8, are involved in apoptosis and thus immune regulation. GZMH^+^ CD8^+^ T cells are expanded in SLE and may promote both caspase-dependent and caspase-independent apoptosis.^40–42^ CASP.8, elevated in both SLE mothers and their neonates, plays key roles in both extrinsic and intrinsic apoptotic pathways.^43 44^ LAMP3 has been implicated in SjD pathogenesis through its inhibition of autophagy and induction of apoptosis.^45^ While a synonymous LAMP3 single nucleotide polymorphism (SNP) has been associated with SLE, further investigation of its functional role is warranted.^46^

Conversely, only two proteins were significantly downregulated in SLE mothers: CD8α and HO-1. The reduced HO-1 expression may be relevant for placental development, as this enzyme regulates angiogenesis, vasculogenesis and trophoblast function.^47 48^ Lower HO-1 expression could thus contribute to oxidative stress, immune dysregulation and adverse pregnancy outcomes in SLE.

The proteins differentially expressed in neonates of SLE mothers largely overlapped with those in maternal samples, including elevated levels of IL-18, CASP.8 and CCL3 and reduced CD8α. These findings further support the maternal-fetal transfer of inflammatory signals and reinforce the strong correlation in protein expression between mothers and their neonates.

When further comparing different antibody-subgroups among SLE mothers and their infants, we found only a few proteins to be significantly differing in expression. Interestingly though, the two proteins, CCL19 and CXCL10, that were significantly upregulated in anti-SSA/SSB-positive SLE mothers and their neonates, respectively, are both IFN-regulated proteins whose expression has been shown to correlate with IFN-signatures and disease activity in SLE patients.^49 50^ Similar to this, in anti-dsDNA-positive mothers, PD-L1 and PD-L2, two of the significantly upregulated proteins, are specific type I IFN-regulated proteins and as such also among the proteins we used to calculate the IFN scores in this study.^20 21^ Notably, CASP.8 was significantly downregulated in anti-SSA/SSB-exposed compared with non-exposed neonates which might explain the decreased CASP.8 levels we found when comparing neonates born to treated with those born to untreated women given that most untreated women were actually anti-SSA/SSB-negative. Still, any described findings based on treatment-specific subgrouping should purely be seen as exploratory, both due to the limited number of women or neonates, respectively, in the different groups and further due to the missing information on maternal disease activity, which might influence both immune protein expression and the need for immunomodulatory treatment. Finally, the increased expression of IL-1α in anti-dsDNA-exposed neonates is not surprising given its role as an alarmin released from damaged or dying cells, which are a general hallmark for SLE, but most importantly collateral to the occurrence of autoantibodies targeting anti-dsDNA and other intracellular antigens.^51 52^

IFN scores—calculated using CXCL10, CXCL11, TRAIL, LAMP3, PD-L1 and PD-L2 expression levels—were significantly elevated in SLE mothers, but not their neonates, which explains the lack of a clearly significant correlation between maternal and neonatal IFN scores observed in this study. Interestingly, the elevated maternal IFN scores were detected regardless of the anti-SSA/B and anti-dsDNA autoantibody status. The lack of differences in IFN scores between anti-Ro/La-positive and -negative and anti-dsDNA-positive and anti-dsDNA-negative SLE mothers, respectively, may be due to the presence of other autoantibodies (eg, anti-Sm, anti-RNP and anti-nucleosome autoantibodies), many of which can form immune complexes that activate type I IFN pathways through mechanisms such as cytokine induction and neutrophil extracellular trap (NET) formation and of which some even have been suggested to be the main drivers of type I IFN activity in SLE.^53–59^

Although previous studies suggested that maternal IFN activation is mirrored in neonates of anti-Ro/SSA-positive mothers,^14^ we found only a slight, non-significant increase in IFN scores in these neonates. This discrepancy may be attributable to maternal immunomodulatory treatment, which has been indicated to dampen neonatal IFN responses more effectively than maternal IFN activation.^14^

In summary, we identified a range of immune-related proteins, particularly pro-inflammatory cytokines and chemokines, that are elevated in pregnant women with SLE and partially reflected in their newborns. We demonstrated that increased IFN pathway activation mainly occurs independently of anti-Ro/SSA, anti-La/SSB and anti-dsDNA autoantibody status, and that maternal treatment may modulate neonatal IFN activation. Our findings suggest that factors beyond anti-Ro/SSA, anti-La/SSB and anti-dsDNA antibodies—such as other autoantibodies and treatment—contribute to maternal and neonatal immune activation in SLE.

The present study has several limitations that should be considered when interpreting the findings. The relatively small cohort and use of historical samples limited the availability of comprehensive clinical data, particularly regarding disease activity throughout pregnancy and detailed treatment information. This limited our ability to account for potential confounding clinical factors and to draw firm conclusions regarding the effects of maternal treatment. Although the relatively small sample size limited the ability to perform adequately powered subgroup analyses, we nevertheless observed differences between the chosen subgroups. In all, these findings suggest an activation of the neonatal immune system in infants born to mothers with SLE irrespective of their specific autoantibody status. Further investigation especially of the relationship between maternal treatment and neonatal immune profiles in SLE pregnancies is needed. Larger prospective studies with comprehensive clinical data and longitudinal follow-up of the children are required to confirm these observations, better understand the underlying mechanisms and determine whether the observed neonatal immune signatures have any clinical relevance.

## Supplementary material

10.1136/lupus-2026-002001 online supplemental file 1

## Data Availability

All data relevant to the study are included in the article or uploaded as supplementary information.
