# Supplementary material for: Protein biomarkers of inflammation in women with SLE and their newborn children
Source: Lupus Sci Med. 2026 Sep 29;13(2):e002001. doi: 10.1136/lupus-2026-002001 (PMC13629874; doi:10.1136/lupus-2026-002001)
Supplement: Supplementary file 1 [file lupus-13-2-s001.pdf]

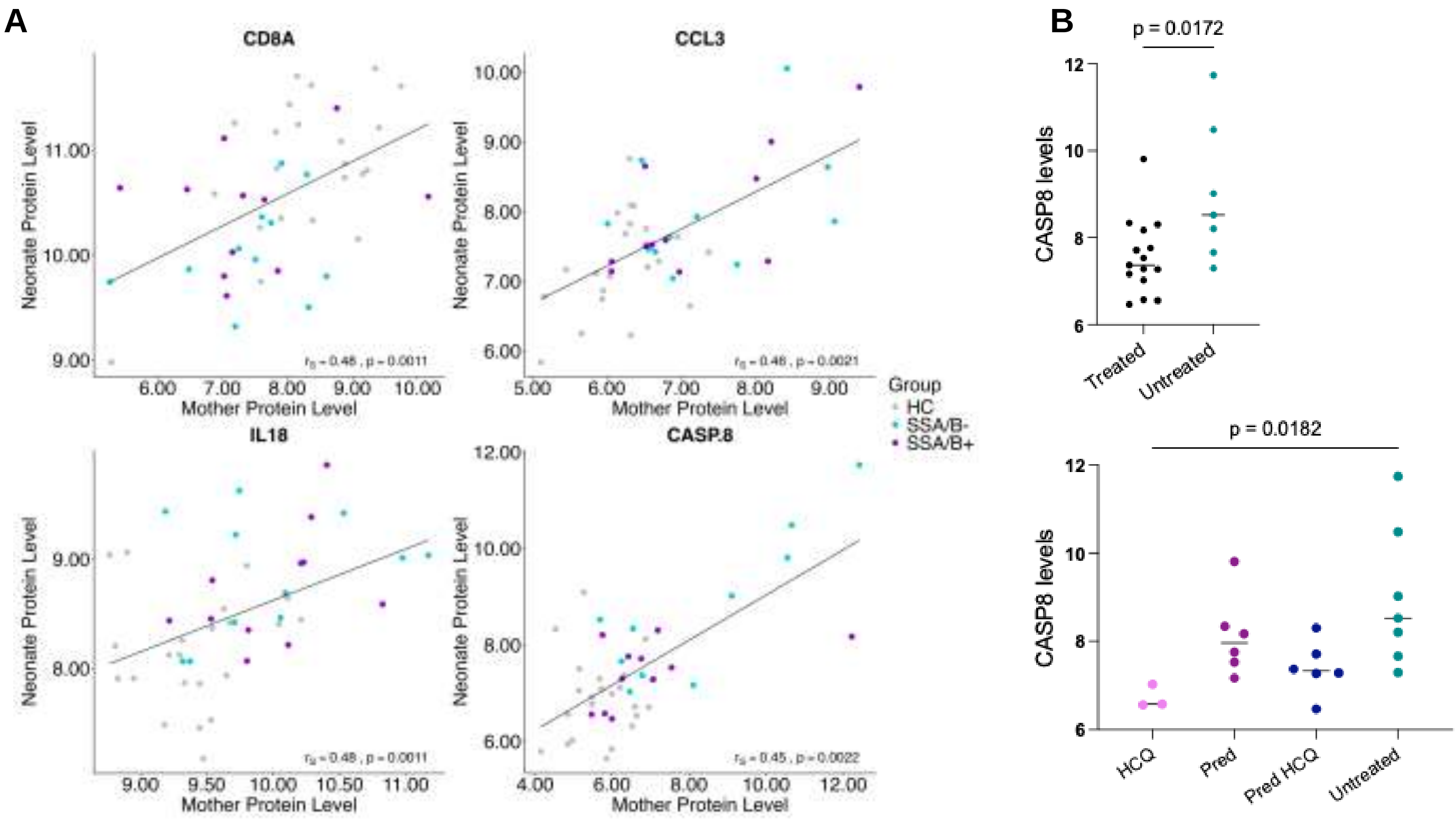

Supplementary Figure 1. Correlating proteins between mothers and neonates.
A) Correlation plots of expression levels of CD8A, CCL3, IL-18 and caspase-8 between maternal and neonatal samples. Spearman's rank correlation coefficients r_S and *p*-values have been calculated and are depicted for all four proteins. Color indicates maternal diagnosis group: grey = healthy controls; blue = SSA/SSB-negative SLE; violet = SSA/SSB-positive SLE.
B) Dot plot depicting neonatal caspase-8 levels in treated versus untreated SLE mothers (upper plot) and untreated versus the different treatment groups (grey = HCQ; violet = Pred; dark blue = Pred+HCQ; lower plot). Statistical analysis was performed using Mann-Whitney U-test for comparison of the treated versus untreated groups (upper panel). For overall comparison between the different treatment groups we used Kruskal-Wallis test followed by Dunn's test. *P*-values less than 0.05 were considered significant and are stated in the plots.

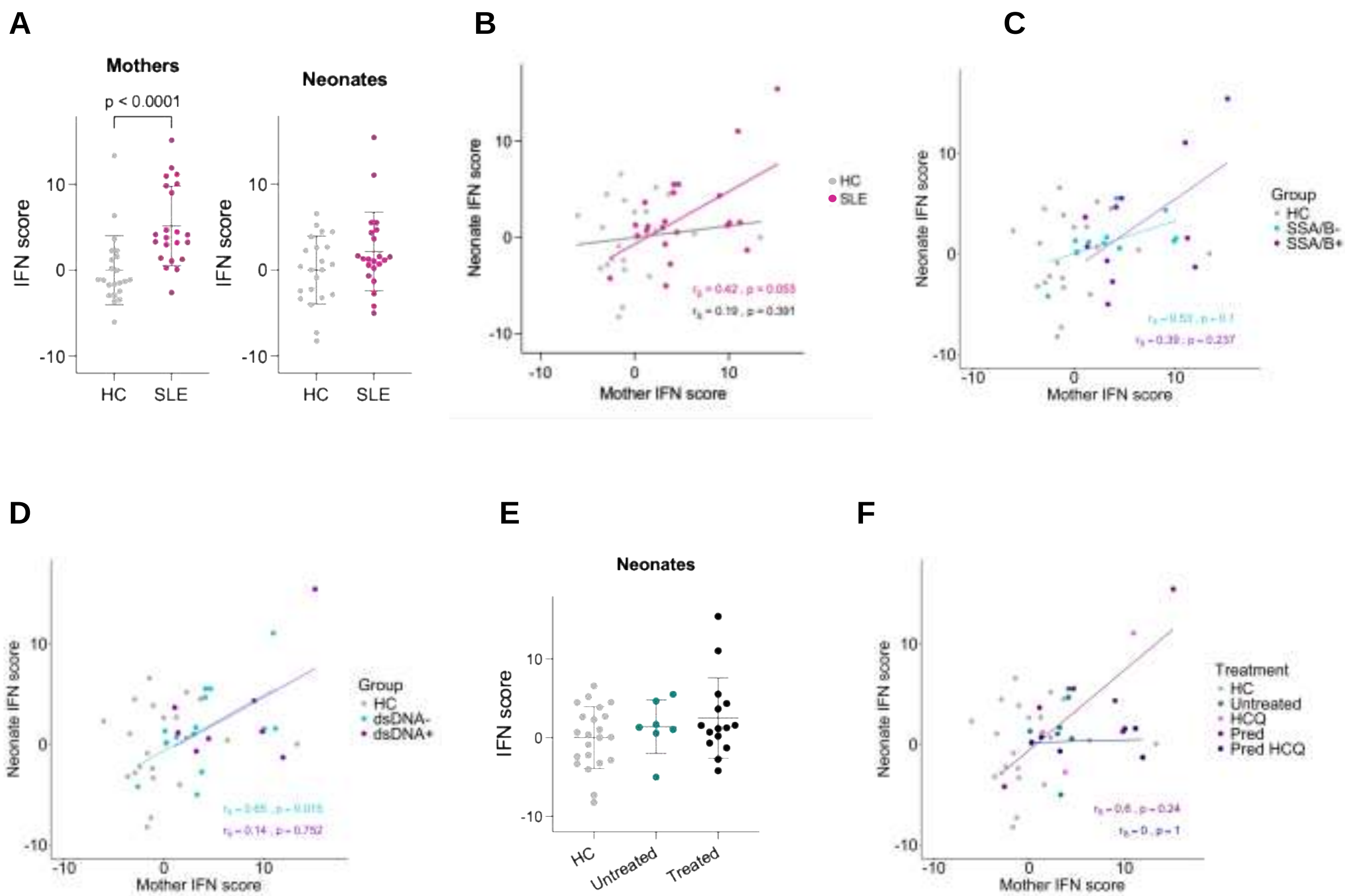

Supplementary Figure 2. Correlation of IFN scores between mothers and neonates.
A) Dot plots depicting IFN scores for healthy control (HC, grey) and SLE mothers (pink, left plot) and their respective neonates (right plot). Statistical analysis was performed using Mann-Whitney U-test and *p*-values less than 0.05 were considered significant and are stated in the plots.
B) Correlation of IFN scores between mothers and their neonates, color-coded for healthy control (HC, grey) and SLE (pink) pairs. Spearman's rank correlation coefficients have been calculated: $r_S = 0.19$ ($p = 0.391$) for HC pairs and $r_S = 0.42$ ($p = 0.053$) for SLE pairs.
C) Same correlation analysis as in A, color-coded by group and according to anti-SSA/SSB autoantibody status: grey = healthy controls; blue = SSA/SSB-negative SLE; violet = SSA/SSB-positive SLE. Spearman's rank correlation coefficients are $r_S = 0.53$ ($p = 0.1$) for the SSA/SSB-negative and $r_S = 0.39$ ($p = 0.237$) for the SSA/SSB-positive pairs.
D) Same correlation analysis as in A, color-coded by group and according to anti-dsDNA autoantibody status: grey = healthy controls; blue = dsDNA-negative SLE; violet = dsDNA-positive SLE. Spearman's rank correlation coefficients are $r_S = 0.65$ ($p = 0.015$) for the dsDNA-negative and $r_S = 0.752$ ($p = 0.752$) for the dsDNA-positive pairs.
E) Dot plot depicting IFN scores for neonates born to healthy control mothers (HC, grey) as well as untreated (green) and treated (black) SLE mothers. Statistical analysis was performed using Kruskal-Wallis test, followed by Dunn's test.
F) Same correlation analysis as in A, color-coded by maternal treatment: grey = healthy controls; light blue = untreated; pink = HCQ; violet = Pred; dark blue = Pred + HCQ. Spearman's rank correlation coefficients are $r_S = 0.6$ ($p = 0.24$) for Pred alone and $r_S = 0$ ($p = 1$) for Pred + HCQ.

Supplementary table 1a. Demographic and clinical characteristics of included neonates grouped according to anti-SSA/SSB autoantibody status

	Neonates born to mothers with rheumatic disease	
	Anti-SSA/SSB+, n=9/11	Anti-SSA/SSB-, n=5/11
**GA at birth** (weeks+days, mean±SD)	38.4.±1.74	39.4±1.14
**Mode of delivery** (%)		
Caesarean section	2 (36.4)	3 (60)
Vaginal	7 (63.6)	2 (40)
**Sex** (%)		
Female	3 (33.3)	3 (60)
Male	6 (66.7)	2 (40)
**Weight** (g, mean±SD)	2994±490.4*	4072±443.8
**Height** (cm, mean±SD)	49.0±2.77	53.0±3.74
**Head circumference** (cm, mean±SD)	35.0±1.63	36.5±1.73

*GA, gestational age; n.a., not applicable; *, p=0.0040*

Supplementary table 1b. Demographic and clinical characteristics of included neonates grouped according to anti-dsDNA autoantibody status

	Neonates born to mothers with rheumatic disease	
	Anti-dsDNA+, n=5/8	Anti-dsDNA-, n=9/14
**GA at birth** (weeks+days, mean±SD)	38.8±1.80	38.8±1.79
**Mode of delivery** (%)		
Caesarean section	3 (60)	2 (36.4)
Vaginal	2 (40)	7 (63.6)
**Sex** (%)		
Female	3 (60)	3 (33.3)
Male	2 (40)	6 (66.7)
**Weight** (g, mean±SD)	3529±532.7	3269.5±862.1
**Height** (cm, mean±SD)	51.0±1.83	50.0±4.24
**Head circumference** (cm, mean±SD)	36.0±1.00	35.4±2.00

GA, gestational age; n.a., not applicable

Supplementary Table 2. Analytes included in the Olink Immuno-Oncology panel.

ADA	CRTAM	IFN-gamma	MIC-A B
ADGRG1	CSF-1	IL-1 alpha	MMP12
ANG-1	CX3CL1	IL-21	MMP7
ANGPT2	CXCL1	IL-35	NCR1
ARG1	CXCL10	IL10	NOS3
CAIX	CXCL11	IL12	PD-L1
CASP-8	CXCL12	IL12RB1	PD-L2
CCL17	CXCL13	IL13	PDCD1
CCL19	CXCL5	IL18	PDGF subunit B
CCL20	CXCL9	IL2	PGF
CCL23	DCN	IL33	PTN
CCL3	EGF	IL4	TIE2
CCL4	FASLG	IL5	TNF
CD244	FGF2	IL6	TNFRSF12A
CD27	Gal-1	IL7	TNFRSF21
CD28	Gal-9	IL8	TNFRSF4
CD4	GZMA	KLRD1	TNFRSF9
CD40	GZMB	LAMP3	TNFSF14
CD40-L	GZMH	LAP TGF-beta-1	TRAIL
CD5	HGF	MCP-1	TWEAK
CD70	HO-1	MCP-2	VEGFA
CD83	ICOSLG	MCP-3	VEGFC
CD8A	IFN-beta	MCP-4	VEGFR-2

Supplementary Table 3a. Differentially expressed proteins – Figure 1B

**Increased in mothers**
CD70
CXCL13
PD-L1
**Increased in infants**
CCL17
CCL20
CD4
CD5
CD8A
FASLG
IL-12
NOS3
TNFRSF4
TNFRSF9
VEGFA

Supplementary Table 3b. Differentially expressed proteins – Figure 1D

**Increased in SLE mothers**	**Increased in healthy mothers**
CX3CL1	HO-1
CD27	CD8A
ICOSLG	
CXCL9	
CCL3	
LAMP3	
CASP-8	
IL-18	
PD-L1	
CRTAM	
PD-L2	
IL-12	
PTN	
TNFRSF9	
PDCD1	
CXCL13	
TNF	
GZMH	
CCL17	
IL-8	
MCP-1	
CCL4	
TNFRSF4	
**Increased in infants born to SLE mothers**	**Increased in infants born to healthy mothers**
IL-18	CD8A
CASP-8	
CCL3	
CXCL11	
